# Psychosocial deprivation, executive functions, and the emergence of socio-emotional behavior problems

**DOI:** 10.3389/fnhum.2013.00167

**Published:** 2013-05-10

**Authors:** Jennifer Martin McDermott, Sonya Troller-Renfree, Ross Vanderwert, Charles A. Nelson, Charles H. Zeanah, Nathan A. Fox

**Affiliations:** ^1^Department of Psychology, University of MassachusettsAmherst, MA, USA; ^2^Department of Human Development and Quantitative Methodology, University of MarylandCollege Park, MA, USA; ^3^Children's Hospital Boston and Harvard Medical School, Harvard Center on the Developing ChildBoston, MA, USA; ^4^Department of Medicine, Tulane University School of MedicineNew Orleans, LA, USA

**Keywords:** event-related potential (ERP), error-related negativity, executive function, conflict monitoring, inhibitory control, institutionalization

## Abstract

Early psychosocial deprivation can negatively impact the development of executive functions (EFs). Here we explore the impact of early psychosocial deprivation on behavioral and physiological measures (i.e., event-related potentials; ERPs) of two facets of EF, inhibitory control and response monitoring, and their associations with internalizing and externalizing outcomes in the Bucharest Early Intervention Project (BEIP; Zeanah et al., 2003). This project focuses on two groups of children placed in institutions shortly after birth and then randomly assigned in infancy to either a foster care intervention or to remain in their current institutional setting. A group of community controls was recruited for comparison. The current study assesses these children at 8-years of age examining the effects of early adversity, the potential effects of the intervention on EF and the role of EF skills in socio-emotional outcomes. Results reveal exposure to early psychosocial deprivation was associated with impaired inhibitory control on a flanker task. Children in the foster care intervention exhibited better response monitoring compared to children who remained in the institution on the error-related positivity (Pe). Moreover, among children in the foster care intervention those who exhibited larger error-related negativity (ERN) responses had lower levels of socio-emotional behavior problems. Overall, these data identify specific aspects of EF that contribute to adaptive and maladaptive socio-emotional outcomes among children experiencing early psychosocial deprivation.

## Introduction

Psychosocial deprivation that occurs in conjunction with institutional rearing can result in perturbations in the development and reactivity of brain regions involved in cognitive processing (Chugani et al., 2001; Eluvathingal et al., 2006; Hanson et al., 2012; Sheridan et al., 2012). Significant impairments have been found amongst children experiencing early psychosocial deprivation on executive functions (EFs; Bos et al., 2009; Loman et al., 2013), skills known to contribute to regulated and goal-directed behavior. In particular, institutional rearing has been linked to perturbations in specific EF skills such as inhibitory control (Colvert et al., 2008a; Pollak et al., 2010; McDermott et al., 2012), conflict resolution (Loman et al., 2013), and working memory (Colvert et al., 2008a; Bos et al., 2009). Across various studies, longer periods of adversity and later age at adoption following psychosocial adversity have both been associated with greater impairment in EFs (Colvert et al., 2008a; Pollak et al., 2010; Merz and McCall, 2011).

In addition to deficits in EF skills, early exposure to psychosocial adversity is associated with elevated rates of neuropsychological problems. These problems are characterized by poor attention as well as dysregulated emotional and behavioral control that may interfere with social relations and academic functioning in childhood (Beckett et al., 2007; Loman et al., 2009). While removal from deprived caregiving environments generally improves developmental outcomes for children (McGoron et al., 2012), continued risk for psychopathology is apparent even after adoption (Colvert et al., 2008b) or placement into high quality foster care (Zeanah et al., 2009), with some problems persisting into adolescence (Colvert et al., 2008b). Recent efforts to identify factors involved in the etiology of these problems among children who experienced early psychosocial deprivation have identified influential biological (McLaughlin et al., 2011) as well as interpersonal factors (McGoron et al., 2012), yet, to our knowledge, no studies have investigated the degree to which deficits in specific EF skills predict maladaptive social outcomes. Given the continued importance of EF skills throughout development, this line of inquiry may be particularly useful in: (1) elucidating cognitive mechanisms that underlie specific socio-emotional problems among previously institutionalized children, and (2) identifying specific areas to target for continued intervention efforts as EF skills.

Two EF skills particularly relevant to risk for psychopathology among children experiencing early psychosocial deprivation are inhibitory control and response monitoring. Inhibitory control is the ability to withhold prepotent actions and suppress irrelevant or distracting information. Response monitoring (also referred to as error monitoring) is the evaluation of one's own actions after they have occurred. This latter skill of response monitoring works in tandem with other EF skills like inhibitory control by signaling the need to adjust behavior to meet task goals. Engagement of inhibitory control and response monitoring (Casey et al., 1997; Bunge and Wright, 2007; Perlman and Pelphrey, 2011) are both guided by areas of the prefrontal cortex (PFC) and anterior cingulate (ACC) and both skills undergo considerable development throughout childhood (e.g., Ridderinkhof et al., 1997; Davies et al., 2004; McDermott et al., 2007; Van Meel et al., 2012). However, the degree to which early experience impacts the emergence and refinement of these skills remains unknown.

Among children experiencing early psychosocial deprivation mixed patterns of inhibitory control performance have emerged. Modest or no differences have been found on basic tests of inhibitory control tracking impulsive responding such as the go/nogo paradigm (McDermott et al., 2012; Loman et al., 2013) and the Knock and Tap test (Pollak et al., 2010). However, measures requiring inhibitory control in the face of distracting stimuli reveal more pronounced deficits with previously institutionalized children exhibiting impairments on Stroop (Colvert et al., 2008a) and flanker tasks (Loman et al., 2013). These patterns suggest that early psychosocial deprivation may differentially influence various brain regions involved in inhibitory control. However, different components of inhibitory control could rely more strongly on specific regions of the PFC and may have variations in developmental patterns. For instance, the go/nogo paradigm assesses delay inhibition, or more specifically, the ability to withhold a prepotent response. This type of inhibitory control involves activation of the ventrolateral PFC (VL-PFC; Durston et al., 2002; Schultz et al., 2004; Goya-Maldonado et al., 2010). In contrast, the flanker paradigm assesses conflict inhibitory control, also referred to as resistance to interference. Conflict inhibitory control is associated with engagement of the dorsolateral prefrontal region (DL-PFC; Casey et al., 2000; Wang et al., 2010; Perlman and Pelphrey, 2011). Brain imaging work suggests that the ability to efficiently engage the DL-PFC may have a more protracted period of development compared to the VL-PFC (e.g., Bunge and Zelazo, 2006). Thus, it is possible that early psychosocial deprivation may differentially influence the development of specific inhibitory control skills or the potential for plasticity in these skills with interventions following early psychosocial deprivation.

Early psychosocial deprivation is also thought to negatively impact the development of response monitoring. The primary measures of response monitoring are two event-related potentials (ERPs): the error-related negativity (ERN; Falkenstein et al., 1991; Gehring et al., 1993) and the error-related positivity (Pe; Falkenstein et al., 1991, 2000). Both components are time locked to subject's responding, however, the ERN is a negative deflection that is maximal at frontocentral sites and generally peaks within the first 100 ms of a response whereas the Pe is a large positive peak with a central-parietal scalp distribution occurring in a later window around 200–500 ms (Falkenstein et al., 2000; Torpey et al., 2012). Functionally, these components are postulated to represent unique processes involved in response monitoring. The ERN is thought to reflect conflict detection associated with response selection or an evaluative signal for action (Coles et al., 2001; van Veen and Carter, 2002; Hermann et al., 2004; Arbel and Donchin, 2009; Roger et al., 2010; Hughes and Yeung, 2011) whereas the Pe represents conscious levels of performance evaluation (Nieuwenhuis et al., 2001). Both the ERN and Pe have been localized to the ACC; however, additional generators have been postulated for the Pe including the anterior insular cortex (Overbeek et al., 2005; Ullsperger et al., 2010; Schroder et al., 2012).

An additional behavioral measure of response monitoring involves the comparison of reaction times (RTs) after correct and incorrect trials. Longer RTs following incorrect trails represent enhanced monitoring via orienting to mistakes (Notebaert et al., 2009), and this RT slowing is postulated to represent efforts to maximize future task performance (Dudschig and Jentzsch, 2009). However, post-error slowing has not been consistently reported across studies and evidence suggests that differences in post-error slowing may be strongly influenced by motivation and personality factors (Luu et al., 2000; Pailing and Segalowitz, 2004).

Recent work examining response monitoring among children experiencing early psychosocial deprivation suggests strong influence of both early psychosocial deprivation and caregiving interventions such as foster care and adoption. McDermott et al. (2012) found that children between 8 and 9 years of age who experienced a high quality foster care intervention following early psychosocial deprivation exhibited stronger response monitoring in the form of a larger ERN compared to children who did not receive the intervention on a go/nogo paradigm. In a study of internationally adopted children, Loman et al. (2013) also found that children who had been in foster care and children who had never been adopted had significantly larger ERN amplitudes compared to children who had previously received institutionalized care on a flanker task. Given that go/nogo and flanker paradigms tap somewhat distinct cognitive skills and neural regions, it is plausible that the impact of early psychosocial deprivation on response monitoring may be more pronounced on flanker as compared to go/nogo tasks. Although these studies reveal deficits in the neural correlates of response monitoring among children experiencing institutional rearing, the potential role of this EF skill in moderating socio-emotional outcomes for these children remains unknown.

The presence of strong EF skills, like inhibitory control or response monitoring, have generally been linked to positive developmental outcomes whereas deficits in EF skills tend to be central components of negative outcomes. In particular, the EF skills of inhibitory control and response monitoring have been strongly implicated in externalizing problems (Olson et al., 2011; Bohlin et al., 2012) such as attention deficit hyperactivity disorder (ADHD; Barkley, 1997; Nigg, 2001; Shiels and Hawk, 2010). Although both externalizing issues and ADHD symptomology are prevalent among children experiencing prolonged psychosocial adversity (Juffer and van Ijzendoorn, 2005; Gunnar et al., 2007; Zeanah et al., 2009), it remains unclear whether differences in EFs may moderation risk for adaptive and maladaptive outcomes among children experiencing early adversity.

Among typically developing children, there is evidence that EFs moderate risk for socio-emotional outcomes. For example, behaviorally inhibited children high in inhibitory control or response monitoring are at increased risk for anxiety issues (McDermott et al., 2009; White et al., 2011) whereas behaviorally inhibited children with high attention shifting skills are at lower risk (White et al., 2011). Such patterns of moderation, along with studies suggesting strong plasticity in EF skills (Rueda et al., 2005), suggest that children at risk for negative socio-emotional outcomes, as in the case of psychosocial deprivation, may benefit from interventions that promote EFs skills. However, no work to date has explored whether EFs moderate socio-emotional outcomes in children experiencing early institutionalized care.

The overarching goals of the current study were to investigate associations among inhibitory control and response monitoring components of EF and the influence of theses skills in social developmental outcomes in a sample of children who experienced early institutionalization and were enrolled in the Bucharest Early Intervention Project (BEIP; see Zeanah et al., 2003 for details). Children in the study were randomized to one of two conditions (1) to be taken out of the institution and placed into foster care (Foster Care Group; FCG) or (2) to remain in institutional care (Care as Usual Group; CAUG). In addition, a typically developing sample of children (Never Institutionalized Group; NIG) was recruited from the community. Behavioral and ERP measures were collected during a flanker task when children were 8 years of age.

Based on a growing literature demonstrating poorer EF skills in children experiencing institutionalized care compared to non-adopted children or children adopted from foster care (see Merz et al., 2013, for a review), it was predicted that children in the CAUG would perform worse than the NIG on the EF measures of inhibitory control and response monitoring whereas children in the FCG would perform at an intermediate level compared to the CAUG and NIG on these measures. Moreover, given the heterogeneity of socio-emotional outcomes exhibited in both the CAUG and FCG at earlier assessments (e.g., Ghera et al., 2009; Zeanah et al., 2009) and the potential for cognitive processes to moderate such outcomes (e.g., McDermott et al., 2009; White et al., 2011), both inhibitory control and response monitoring skills were predicted to moderate associations between early experience and socio-emotional outcomes for all groups such that better EF skills would be associated with lower rates of socio-emotional problems.

## Materials and methods

### Participants

**Figure 1:**
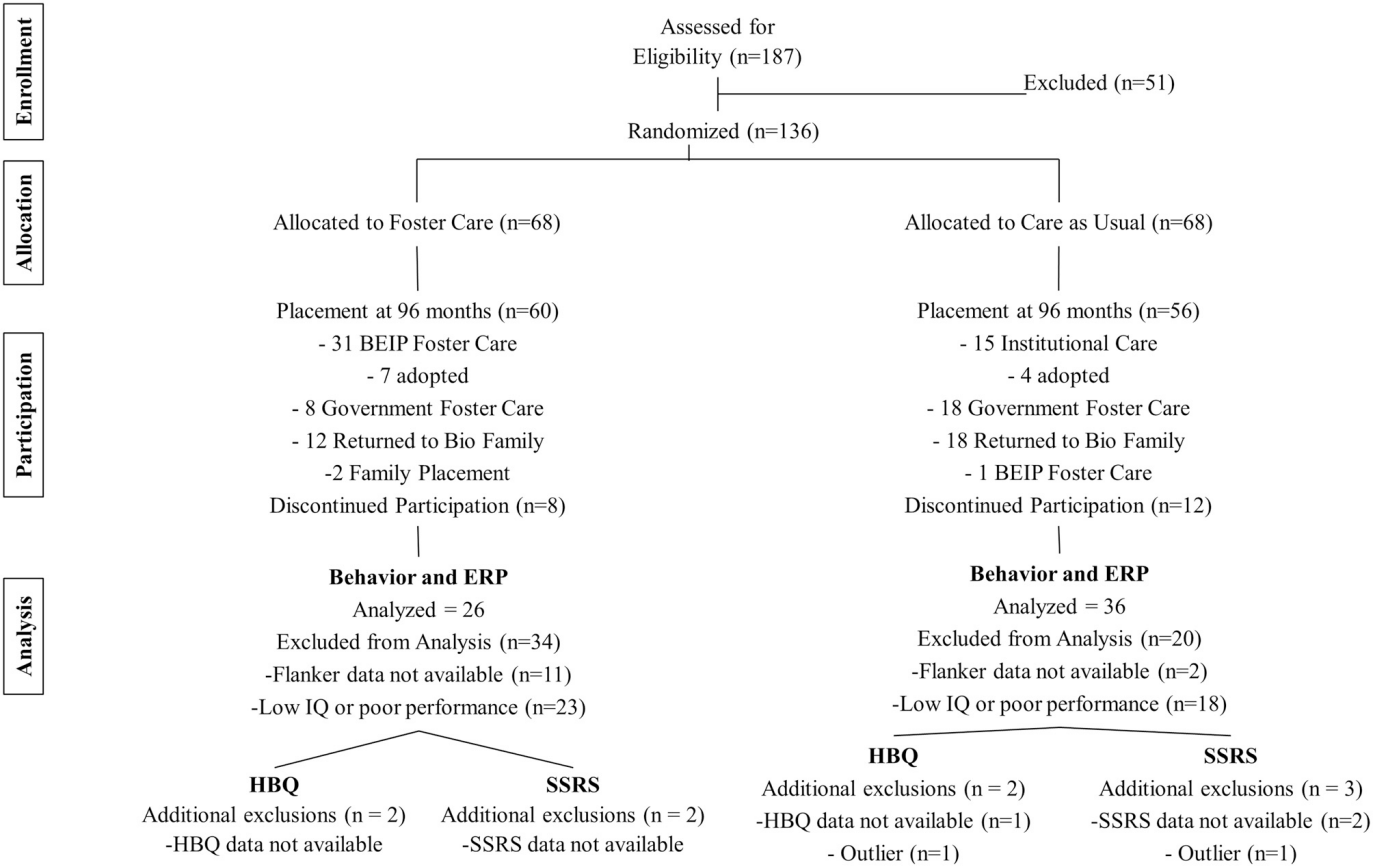
**Group assignment over time: current status at 8 years of age**.

The sample was comprised of 136 children, abandoned at birth and placed into institutional care in Bucharest, Romania who were part of the BEIP. At 8 years, 49 CAUG (25 female), 54 FCG (28 female), and 47 NIG (26 female) children remained in the study and completed the Flanker task that is the subject of this paper. The mean age of test was 104.79 (*SD* = 8.27) months for the CAUG, 104.65 (*SD* = 12.98) months for the FCG, and 100.83 (*SD* = 9.14) months for the NIG. Figure 1 presents a Consort Diagram for the sample at 8 years of age. Although many of the institutionalized children at age 8 were no longer in their original randomized placement, the data to be presented in this paper uses an intent-to-treat approach such that data are analyzed using a child's initial placement.

The University Institutional Review Boards of the principal investigators (Fox, Nelson and Zeanah) and the University of Bucharest, Romania approved the study protocol. Romanian law dictated that consent be given by the local Commission on Child Protection for each child participant who lived in their sector of Bucharest. Electrophysiology assent was obtained from each caregiver who accompanied a child to the visit.

### Flanker task

The flanker task assesses children's ability to respond to a central target in the context of distracting stimuli. For this study the target stimuli consisted of right or left facing arrows. Children were instructed to respond as quickly and correctly as possible via button press to indicate the direction of the middle arrow (right or left). Congruent trials consisted of stimuli all in the same direction (> > > > > or < < < < <), whereas incongruent trials had the central target in a row of stimuli facing in the opposite direction as the flanking stimuli (< < > < < or > > < > >). A practice block of 20 trials was presented to familiarize children with the stimuli and button box. The test trials consisted of equal numbers of congruent and incongruent trials presented in a pseudo random order across two test blocks of 80 trials each for a total of 160 test trials.

Trials began with the presentation of a warning cue (^*****^) for 500 ms, followed by a blank screen and then the presentation of the target display for 1000 ms and then another a blank screen for 500 ms. Children were required to respond within 1500 ms of the presentation of the target array. Task difficulty level was controlled by varying the presentation rate of the flanker stimuli. Based upon participant accuracy across 10 trials, stimulus presentation time sped up (7 or more correct responses), slowed down (5 or less correct responses), or remained the same (6 correct responses). This manipulation resulted in an overall average error of commission rate of ~60%. Stimulus presentation was controlled by computer software (Cognitive Activation System; CAS, James Long Company, Caroga Lake, NY, USA) run on an IBM PC on which the flanker task was programmed. Measures of response time and response accuracy per trial were directly recorded by CAS program software.

### Electroencephalogram (EEG)

EEG was collected via a lycra Electro-Cap (Electro-Cap International Inc., Eaton, OH, USA) according to the 10–20 system from the following sites: F3, F4, Fz, C3, C4, P3, P4, Pz, O1, O2, T7 and T8 and the right and left mastoids. Site Cz served as reference and Afz served as ground. Impedances were kept at or below 10 kΩ. Eye movement was tracked via electrooculogram (EOG) collected from a pair of Beckman mini-electrodes with one placed above and one below the left eye. Electrophysiological signals were amplified at 5000 for EEG and 2500 for EOG. Bandpass filters of 0.1–100 Hz were applied with custom bioamplifiers from the James Long Company (Caroga Lake, NY, USA) and data were digitized at 512 Hz. For ERP analysis a 30-Hz lowpass filter was applied, epochs containing signals exceeding ±200 uV were excluded and EOG artifact was regressed. An average mastoid configuration was used to re-reference the data and baseline correction for individual averages was calculated with the 200–100 ms prior to response. Averages were calculated for correct and incorrect trials to examine patterns of the ERN and Pe. Peak amplitudes for both the ERN and Pe were assessed at the midline electrodes (Fz, Cz, Pz) and time-locked to button press. The ERN was examined in the windows of −50 to 100 ms where as the Pe was assessed in the window of 110–210 ms.

### Health and behavior questionnaire (HBQ, MacArthur)

For the present paper, four behavioral subscales of the HBQ were used: internalizing behaviors, externalizing behaviors, ADHD symptoms, and social withdrawal. The internalizing scale is comprised of items related to depression and overanxious behaviors. The externalizing scale consists of measures of oppositional defiance, conduct problems, overt hostility, and relational aggression. The ADHD scale measures inattention and impulsivity. Finally, the social withdrawal scale is comprised of asocial behavior with peers and social inhibition. Each participant's primary teacher completed the HBQ.

### Social skills rating system (SSRS, pearson assessments)

The SSRS assesses three broad scales: problem behaviors, social skills, and academic competence. The problem behaviors scale focuses on three types of issues that can interfere with social development: externalizing, internalizing, and hyperactivity. The social skills scale assesses aspects of positive social behavior such as cooperation, empathy, assertion, self-control, and responsibility. Academic competence reflects a child's performance on reading, mathematics, global cognitive ability, motivation, and parent support. The SSRS was completed by each participant's primary teacher.

#### IQ

At 8 years of age, IQ was assessed in the BEIP laboratory using the Wechsler Intelligence Scale for Children (WISC-IV; Wechsler, 2003). The WISC-IV uses 10 subtests to assess intellectual functioning in four domains: verbal comprehension, perceptual reasoning, working memory, and processing speed. In addition, a full-scale IQ composite score is calculated based on the 10 subtest scores, scaled for age. The four subscale scores and full-scale IQ scores were used in the present analyses. The IQ data were previously reported in Fox et al. (2011). Trained and reliable Romanian psychologists administered all of the IQ scales.

### Participant inclusion

To verify capacity to complete the flanker task, children who scored less than 70 on the WISC or had less than 60% accuracy on congruent trials were excluded from analysis (23 CAUG, 18 FCG, 4 NIG) [χ^2^_(2, *N* = 150)_ = 17.316, *p* < 0.001]. The final sample for behavioral analysis included 26 (15 female) CAUG children, 36 (19 female) FCG children, and 43 (25 female) NIG children. For ERP analysis, children with fewer than eight usable trials for the response-locked ERN (1 NIG) (Olvet and Hajcak, 2009) and five additional NIG children who only completed the task behaviorally were excluded from analysis. The final sample for ERP analysis included 26 (15 female) CAUG children, 36 (19 female) FCG, and 37 (24 female) NIG children. Finally, for the social outcome moderation analyses, four children were removed from the HBQ analyses (2 IG, 1 FCG, 1 NIG) and six children were removed from the SSRS analyses due to missing data (2 IG, 2 FCG, 2 NIG). An additional three children with extreme (more than 3 SD from the group mean) scores on the HBQ and SSRS scales were excluded (1 FCG, 2 NIG). The final moderation samples included 24 (14 female) CAUG children, 34 (17 female) FCG, and 34 (22 female) NIG children for HBQ analyses and 24 (14 female) CAUG children, 33 (16 female) FCG, and 33 (21 female) NIG children for SSRS analyses.

## Results

### Statistical procedures

To assess behavioral responses a series of repeated measures ANOVAs were used with Greenhouse-Geisser corrections applied as necessary. Participant group (CAUG, FCG, NIG) served as a between-subjects factor and flanker trial type (congruent vs. incongruent) served as a within-subjects factor.

To assess group differences in the ERN and Pe, separate ANOVAs were conducted with participant group (CAUG, FCG, NIG) as the between-subjects factor. Additionally, to assess the influence of participant group and the ERN on children's socio-emotional outcomes (HBQ and SSRS scales) a series of linear regressions were run. To address potential mulitcollinearity and clarify in analysis interpretation, interaction terms were standardized and mean centered. The three groups of children (CAUG, FCG, NIG) were effect coded into two variables in order to exhaust all possible comparisons. Group and the flanker-related variables were entered first followed by the interaction terms to look for moderation effects of accuracy or neural reactivity (ERN/Pe). Significant Group by flanker-related variable moderation effects were probed by follow-up 3 Group (IG, FCG, NIG) ×2 ERN Size (large, small) ANOVAs.

### Behavior

#### Accuracy

A main effect was found for trial type [*F*_(1, 102)_ = 361.96, *p* = 0.00] with more accurate responding on congruent (*M* = 81.94%, *SD* = 9.05) compared to incongruent trials (*M* = 44.60%, *SD* = 18.88). This main effect was qualified by an interaction of trial type and group [*F*_(2, 102)_ = 4.23, *p* = 0.02]. Follow-up tests revealed that the groups differed in accuracy rates on incongruent trials [*F*_(2, 104)_ =6.28, *p* = 0.00] such that the CAUG and FCG were significantly less accurate on incongruent trials than children in the NIG (*p*'s = 0.02). The CAUG and FCG did not differ in their accuracy rates.

#### Reaction time

Congruency effects were analyzed by comparing RT on correct congruent and correct incongruent trials. A main effect for trial emerged [*F*_(1, 102)_ = 112.27, *p* = 0.000] such that children responded faster on congruent (*M* = 694 ms, *SD* = 107 ms) as compared to incongruent trials (*M* = 776 ms, *SD* = 135 ms). Both the main effect of trial along with a main effect for group [*F*_(2, 102)_ = 3.70, *p* = 0.028] were qualified by an interaction between trial type and group [*F*_(2, 102)_ = 3.44, *p* = 0.036]. Follow-up tests revealed group differences for RTs on congruent trials [*F*_(2, 104)_ = 6.21, *p* = 0.003]. Specifically, children in the CAUG and FCG groups had slower congruent trial RTs than children in the NIG (*p*'s < 0.05). The CAUG and FCG groups did not differ in their overall RTs on congruent trials (see Table 1).

**Table 1:** **Descriptive statistics**.

	**NIG**	**CAU**	**FCG**
Age (months)	8.31 (0.27)	8.49 (0.44)	8.68 (0.35)
**BEHAVIOR**			
Overall accuracy (%)	67.5 (11.10)	58.9 (8.00)	61.3 (10.13)
Congruent trials	83.0 (9.51)	78.4 (8.13)	82.3 (8.65)
Incongruent trials	52.1 (16.345)	39.5 (17.23)	39.4 (20.15)
Reaction time (ms)	702 (115)	777 (97)	739 (115)
Congruent trials	653 (100)	736 (89)	713 (113)
Incongruent trials	754 (146)	822 (121)	768 (126)

Post-error RT slowing was assessed by comparing RTs after correct trials to RTs following errors of commission. A main effect for trial emerged [*F*_(1, 102)_ = 112.27, *p* = 0.00] with faster responding after correct trials (*M* = 731 ms, *SD* = 117 ms) compared to errors of commission (*M* = 750 ms, *SD* = 117). Additionally, main effect of group [*F*_(2, 102)_ = 3.70, *p* = 0.028] revealed differences in general processing speed across the groups. The follow-up analysis revealed that collapsed across trial type the CAUG responded significantly slower than the NIG (*p* = 0.018) whereas the FCG did not significantly differ in their response speed from either the CAUG or NIG. No interactions between reaction and group emerged for post-error RT slowing.

### Event-related potentials

#### ERN

**Figure 2:**
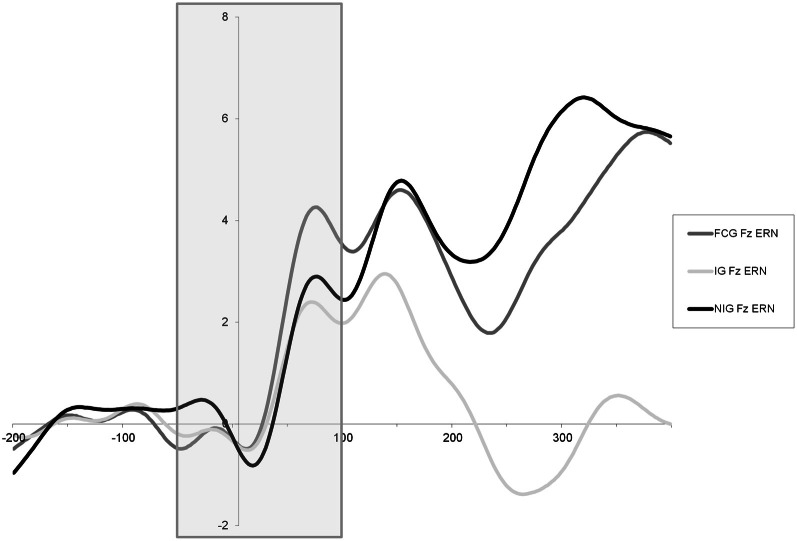
**Response-locked ERN waveform at Fz.** ERN time window is outlined.

Main effects for trial [*F*_(1, 98)_ = 18.138, *p* = 0.000] and site [*F*_(2, 196)_ = 6.027, *p* = 0.007] as well as a trial × site interaction [*F*_(2, 196)_ = 20.906, *p* = 0.000] emerged on a repeated measures ANOVA that revealed larger ERN amplitudes on incorrect trials that were maximal at site Fz. In order to examine group differences in the ERN peak amplitude at Fz, a 3 Group (CAUG, FCG, NIG) One-Way ANOVA was performed. Results indicated that there were no significant group differences in ERN peak amplitude [*F*_(2, 96)_ = 1.310, *p* = 0.275] (Figure 2). Additionally, among the FCG, ERN peak amplitude was not correlated with percent of life spent in institutionalized care [*r*_(34)_ = 0.020, *p* = 0.906].

#### Pe

A repeated-measures ANOVA revealed main effects for trial [*F*_(1, 98)_ = 12.215, *p* = 0.001], site [*F*_(2, 196)_ = 53.014, *p* = 0.000] that were qualified by a trial × site interaction trial [*F*_(2, 196)_ = 37.786, *p* = 0.000] showing that Pe amplitude was larger on incorrect trials and greatest at sites Cz and Pz. Separate One-Way ANOVAs were used to examine group differences (CAUG, FCG, NIG) in Pe peak amplitude at Cz and Pz. Main effects for group emerged at site Cz [*F*_(2, 96)_ = 6.925, *p* = 0.002] and Pz [*F*_(2, 96)_ = 5.621, *p* = 0.005] and follow-up tests reveal that at site Cz the NIG displayed larger Pe responses compared to the CAUG (*p* = 0.001) and the FCG (*p* = 0.057) with similar patterns emerging at site Pz (*p* = 0.009 and *p* = 0.030, respectfully). Pe peak amplitude was not correlated with percent of life spent in institutionalized care among the FCG at site Cz [*r*_(34)_ = −0.117, *p* = 0.364] or Pz [*r*_(34)_ = −0.040, *p* = 0.759].

### Effects of executive function measures on socio-emotional outcomes

Separate multiple regression analyses were used to test if the EF measures of inhibitory control (i.e., accuracy, RT) or response monitoring (i.e., ERN, Pe, post-response RT) predicted socio-emotional outcomes on the HBQ (externalizing-ADHD, internalizing, and social withdrawal) and the SSRS (academic competence, social skills, and problem behaviors).

### Inhibitory control

The analyses examining potential moderating effects of inhibitory control variables (accuracy and RT) for both the HBQ and SSRS outcome variables failed to reach significance.

### Response monitoring

#### HBQ

**Figure 3:**
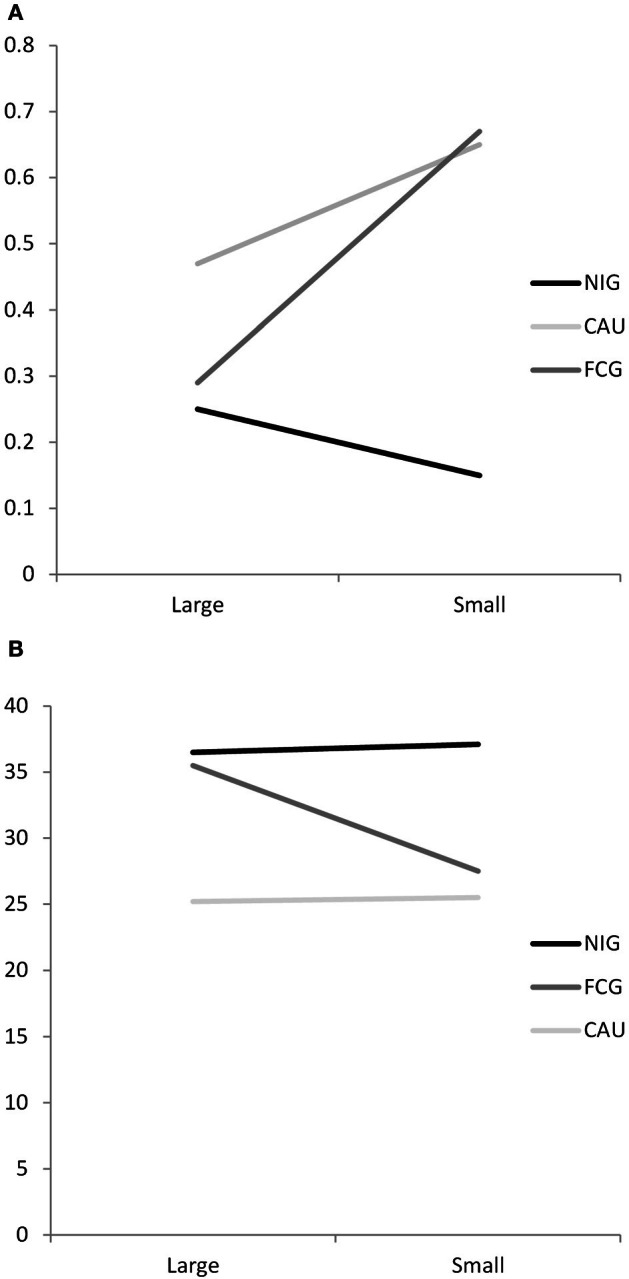
**Moderation analyses of Group × ERN for (A) externalizing-ADHD behaviors as measured by the HBQ (B) academic competence as measured by the SSRS**.

A significant Group × ERN moderation predicted externalizing-ADHD behaviors [β = 0.292, *t*_(86)_ = 2.781, *p* = 0.007]. This moderation was probed by a 3 Group (CAUG, FCG, NIG) ×2 ERN median split (large, small) ANOVA. A main effect for Group [*F*_(2, 86)_ = 7.997, *p* = 0.001] emerged, but was qualified by a Group × ERN interaction [*F*_(2, 86)_ = 4.699, *p* = 0.012]. *Post-hoc* tests revealed that within the FCG, children with smaller ERNs exhibited significantly more externalizing-ADHD behaviors than children with large ERNs (see Table 2, Figure 3A). This pattern was further supported by the finding that among children with small ERN responses, FCG and CAUG exhibited significantly more externalizing-ADHD behaviors than NIG children. Children with large ERN responses showed a similar number of externalizing-ADHD problems, regardless of participant group.

**Table 2:** **ANOVA moderation means**.

	**NIG**		**CAU**		**FCG**	
	**Large ERN**	**Small ERN**	**Large ERN**	**Small ERN**	**Large ERN**	**Small ERN**
**HBQ**						
Externalizing-ADHD	0.25 (0.33)	0.15 (0.16)	0.47 (0.30)	0.65 (0.39)	0.29 (0.27)	0.67 (0.37)
**SSRS**						
Academic competence	36.5 (5.8)	37.1 (5.9)	25.2 (7.0)	25.5 (6.4)	35.5 (5.8)	27.5 (7.5)

The Group × ERN moderation analyses examining internalizing problems and social withdrawal failed to reach significance. Additionally, the Group × Pe moderation analyses did not reach significance for any of the HBQ outcomes variables.

#### SSRS

A significant Group × ERN moderation predicted academic competence [β = −0.325, *t*_(84)_ = −3.914, *p* < 0.001] and social skills scores [β = −0.225, *t*_(84)_ = −2.418, *p* = 0.018]. These moderations were probed by a 3 Group (CAUG, FCG, NIG) ×2 ERN median split (large, small) ANOVA. For academic competence, a main effect of group emerged [*F*_(2, 84)_ = 23.972, *p* < 0.001], but was qualified by a Group × ERN interaction [*F*_(2, 84)_ =4.936, *p* = 0.009]. *Post-hoc* tests indicated in the FCG group, children with a large ERN response exhibited more academic competence than FCG children with a small ERN (see Table 2, Figure 3B). This pattern was further supported by the finding that FCG children in the large ERN group had the same level of academic competence as the NIG and significantly more academic competence than the CAUG. Furthermore, FCG children with a small ERN response showed academic competence similar to CAUG children and significantly less academic competence than NIG children. The follow-up analyses for the social skills failed to reach significance.

The Group × ERN moderation analyses for problem behaviors outcome failed to reach significance.

#### Pe

At site Cz a significant Group × Pe moderation predicted academic competence [β = −0.252, *t*_(86)_ = −2.803, *p* = 0.006] and social skills [β = −0.227, *t*_(86)_ = −2.356, *p* = 0.021]. These moderations were probed by separate 3 Group (CAUG, FCG, NIG) ×2 Pe median split (large, small) ANOVAs. For academic competence, a main effect for Group [*F*_(2, 84)_ = 23.849, *p* = 0.000] emerged that was qualified by a Group × Pe interaction [*F*_(2, 84)_ = 6.013, *p* = 0.004]. *Post-hoc* tests revealed that within the NIG, children with smaller Pe responses exhibited significantly less academic competence than children with large Pe responses. Among children with large Pe responses, FCG and CAUG children exhibited significantly less academic competence than NIG children (*p*'s = 0.000). However, among children with low Pe responses, both the NIG (*p* = 0.015) and FCG (*p* = 0.029) exhibited higher academic competence than CAUG children.

For social skills the Group × Pe interaction failed to reach significance. The moderation analyses for the problem behaviors outcome also failed to reach significance.

## Discussion

The current study examined the impact of early psychosocial deprivation on the EF skills of inhibitory control and response monitoring. The potential influence of these skills on socio-emotional outcomes was also investigated. Two sets of results emerged. First, impairments in inhibitory control, but not response monitoring, were noted among children who experienced psychosocial deprivation. Second, neural markers of response monitoring were enhanced among children in the foster care intervention and moderated associations between deprived caregiving experience and the expression of socio-emotional behavior problems in childhood. Combined, these results highlight the multi-faceted impact of early psychosocial deprivation on the development of cognitive processing skills and behavioral functioning in childhood.

Although all children performed the flanker task as expected, with standard patterns of increased accuracy and faster RTs on congruent as compared to incongruent trials, there were several notable group differences. Both the CAUG and FCG were less accurate than the NIG children on incongruent trials. Poor performance on incongruent trials suggests that the nature of inhibitory control deficits that continue into childhood among children experiencing early psychosocial deprivation might be strongly influenced by conflict inhibition rather than delay inhibition. This notion is supported by work that has shown impairments in previously institutionalized children on a Stroop task that involves similar levels of cognitive conflict as the flanker (Colvert et al., 2008a) and also by the lack of impulsivity problems exhibited by these children in go/nogo tasks (McDermott et al., 2012; Loman et al., 2013).

Whereas the current study found group differences on the cognitively demanding incongruent trials of the flanker task, Loman et al. (2013) found general deficits in behavioral accuracy on a flanker task among a group of previously institutionalized children. Both task structure and participant age, may have contributed to performance variations found between the two studies. Namely, the current study employed a version of the flanker paradigm that dynamically adjusted throughout the task and thus may have magnified participant focus and minimized potential differences in errors of omission. Additionally, the children in the current study were also slightly younger than the children in the study by Loman et al. (2013). Because the flanker task is a cognitively challenging task even among typically developing children of this age range (e.g., Ridderinkhof et al., 1997) it is plausible that improvements in incongruent trial accuracy may emerge with age among the previously institutionalized children in the BEIP sample.

Deficits in processing speed also emerged for both the CAUG and FCG children compared to never institutionalized children. Interestingly, the differences were significant only for congruent trials. This pattern may reflect general deficits in processing speed and corresponds to recent work demonstrating alterations among children experiencing early psychosocial deprivation in white matter structure postulated to underlie processing speed (Hanson et al., 2013). Alternatively, it may indicate different task strategies among the groups as the FCG RTs on incongruent trials were more in line with RTs by the NIG compared to the CAUG. Thus, the slowed response among the FCG children on the “easier” congruent trials could result from a performance strategy to maximize accuracy outcomes rather than a standard deficit in processing speed. Further longitudinal work is needed to illuminate whether processing speed differences are maintained or remediated over time and among children in the foster are intervention as processing speed capacity early in life is strongly associated with later cognitive function (Rose et al., 2012).

Although recent work has emphasized the impact of psychosocial deprivation on sustained attention in middle childhood (McDermott et al., 2012; Loman et al., 2013), the current data support the premise of multiple aspects of cognitive impairment depending upon the nature of the cognitive task and the underlying neural regions that it taps. For instance, the ability to execute delay aspects of inhibitory control that rely on VL-PFC, does not guarantee developmentally appropriate mastery of conflict inhibitory control that depend more heavily upon DL-PFC. It is likely the case that the dynamically adjusting version of the flanker task employed in the current study was challenging enough to expose continuing difficulties in the realm of conflict inhibitory control within the BEIP children who experienced early psychosocial deprivation.

A key group difference emerged on the Pe measure of response monitoring such that children experiencing early adversity exhibited diminished neural processing of errors on this component compared children who never experienced early adversity. This result is in line with findings from the study by Loman et al. (2013) in which internationally adopted children exhibited a reduced Pe response. Although this component has been reported to be prominent in children (Torpey et al., 2012), it remains unknown what factors influence the stability of the Pe across contexts or throughout childhood. This is one of the first studies to report associations between Pe amplitude and outcomes among children as children in the NIG with large Pe responses had the highest ratings of academic competence. Given its role in error awareness and orienting to errors, additional work to determine what factors contribute to stronger Pe responses in children are warranted.

Although group differences were not found on the ERN, this component also moderated socio-emotional outcomes for children in the foster care intervention. Specifically, among the FCG a larger ERN response appeared to function as a protective factor as it was linked to in lower rates of externalizing-ADHD and higher academic competence. The opposite pattern of outcomes was found among FCG children with a small ERN response. In contrast, the ERN was not influential for children in the CAUG or NIG. Namely, children in the CAUG had elevated rates of socio-emotional issues and the NIG had lower rates of socio-emotional issues regardless of the magnitude of their ERN response.

The finding of larger ERN responses being linked to more adaptive outcomes among the FCG corresponds to other work examining the ERN and socio-emotional outcomes. Specifically among young children, a larger ERN response is generally adaptive (Meyer et al., 2012) whereas a smaller ERN response has been associated with increased ADHD and externalizing rates and risk for substance use (e.g., Stieben et al., 2007; Euser et al., 2012; Geburek et al., 2012). Further work is needed to determine whether this component indexes awareness and attention toward task performance and/or increased affective relevance of performance outcomes in the FCG. It will also be imperative to determine what aspect of the foster care intervention influences the development of the ERN response and whether strong response monitoring continues to function in an adaptive fashion among the FCG children over time.

Interestingly, the ERN did not modulate risk for internalizing issues in this sample. Emerging work suggests that the standard association between the ERN and increased risk for anxiety (Olvet and Hajcak, 2008) may not be evident across development, as young children with a large ERN response have decreased risk for anxiety problems (Meyer et al., 2012). In children experiencing early psychosocial adversity, emotion regulation issues that result in risk for internalizing problems may continue to increase with age or may be mitigated by a different set of cognitive factors not covered in the current study. Overall the group differences in the current study are a result of a conservative approach to examining the effects of early experience amongst the BEIP sample.

In sum, the current study reveals that psychosocial deprivation negatively impacts the development of conflict inhibitory control with effects lasting through early childhood. Response monitoring skills are similarly impacted by early psychosocial deprivation, however, certain facets of response monitoring are remediated by a foster care intervention. Given work in young children demonstrating links between responsive caregiving can specific aspects of EF (Bernier et al., 2010, 2012) as well as evidence from intervention work in preschool aged children linking enhanced caregiving and improved cognitive control (Bruce et al., 2009), it is plausible that caregiving impacts both neural development underlying error processing as well as performance motivation.

The current data also highlight that the degree of plasticity in certain cognitive skills such inhibitory control or response monitoring may occur over a protracted time period, there is a subset of children from the FCG exhibited a large ERN and more adaptive socio-emotional outcomes. It remains to be determined the mechanism through which some children in the FCG developed a larger ERN response than others. To our knowledge, this is the first paper to demonstrate that response monitoring moderates associations between early psychosocial deprivation, foster care intervention, and socio-emotional outcomes. Future work is needed to explore specifically which aspects of caregiving interactions impact changes in neural development underlying EF and socio-emotional outcomes.

### Conflict of interest statement

The authors declare that the research was conducted in the absence of any commercial or financial relationships that could be construed as a potential conflict of interest.
